# Maturation of type I and type II rat vestibular hair cells *in vivo* and *in vitro*

**DOI:** 10.3389/fcell.2024.1404894

**Published:** 2024-06-04

**Authors:** Mireia Borrajo, David Sedano, Aïda Palou, Víctor Giménez-Esbrí, Alejandro Barrallo-Gimeno, Jordi Llorens

**Affiliations:** ^1^ Departament de Ciències Fisiològiques, Universitat de Barcelona (UB), Hospitalet de Llobregat, Catalunya, Spain; ^2^ Institut de Neurociències, Universitat de Barcelona (UB), Barcelona, Catalunya, Spain; ^3^ Institut d’Investigació Biomèdica de Bellvitge (IDIBELL), Hospitalet de Llobregat, Catalunya, Spain

**Keywords:** vestibular sensory hair cell, type I and type II hair cells, osteopontin, calretinin, contactin-associated protein 1 (CASPR1), plasma membrane calcium-transporting ATPase 2 (PMCA2), SRY-box transcription factor 2 (SOX2), 3D culture

## Abstract

Vestibular sensory epithelia contain type I and type II sensory hair cells (HCI and HCII). Recent studies have revealed molecular markers for the identification of these cells, but the precise composition of each vestibular epithelium (saccule, utricle, lateral crista, anterior crista, posterior crista) and their postnatal maturation have not been described in detail. Moreover, *in vitro* methods to study this maturation are not well developed. We obtained total HCI and HCII counts in adult rats and studied the maturation of the epithelia from birth (P0) to postnatal day 28 (P28). Adult vestibular epithelia hair cells were found to comprise ∼65% HCI expressing osteopontin and PMCA2, ∼30% HCII expressing calretinin, and ∼4% HCII expressing SOX2 but neither osteopontin nor calretinin. At birth, immature HCs express both osteopontin and calretinin. P28 epithelia showed an almost adult-like composition but still contained 1.3% of immature HCs. In addition, we obtained free-floating 3D cultures of the epithelia at P1, which formed a fluid-filled cyst, and studied their survival and maturation *in vitro* up to day 28 (28 DIV). These cultures showed good HC resiliency and maturation. Using an enriched medium for the initial 4 days, a HCI/calretinin+-HCII ratio close to the *in vivo* ratio was obtained. These cultures are suitable to study HC maturation and mature HCs in pharmacological, toxicological and molecular research.

## 1 Introduction

The vestibular system contains five sensory epithelia, comprised of two maculae, utricle, and saccule, and three cristae: lateral, anterior, and posterior. The transducing cells in these epithelia are named hair cells (HC) and belong to one of two types, I and II (Wersäll, 1956). Type I HC (HCI) have an amphora-like shape with a narrow neck and are encased by the calyx, a unique kind of terminal formed by the post-synaptic afferent (Lysakowski et al., 2011). This arrangement contributes to a kind of transmission, named nonquantal, that reduces its latency (Govindaraju et al., 2023). Type II HCs (HCII) are more cylindrical in their bodies, often show basal extensions and are contacted by afferent terminals with standard bouton shapes (Pujol et al., 2014). Besides morphology, these two types of cells differ also in many molecular and physiological characteristics (Rüsch et al., 1998; Eatock and Songer, 2011; McInturff et al., 2018), as well as in their sensitivity to toxic insults (Lindeman, 1969; Maroto et al., 2021; Maroto et al., 2023).

One open question of interest is that of how many cells of each type are present in each type of sensory organ. Although there have been thorough attempts to answer this question in the past (Lindeman et al., 1981; Desai el al., 2005a; Desai el al., 2005b) the use of selective molecular markers and confocal microscopy may resolve the remaining uncertainties. As for markers, HCII have been long known to express calretinin in rats and mice (Dechesne et al., 1991; Desai et al., 2005a; Desai el al., 2005b), but some doubts remain of whether some HCI may also express it, or whether all HCII do (Desai et al., 2005a; Desai el al., 2005b; McInturff et al., 2018). Calretinin also labels afferents that only form calyx terminals and that characterize the central part of the sensory organs (Desmadryl and Dechesne, 1992), but their morphology clearly differentiate these calyces from HCII. Other proposed HCII markers are annexin A4 (ANXA4) and microtubule-associated protein tau (MAPT) (McInturff et al., 2018). Also, in adult mice, expression of the transcription factor SRY-Box Transcription Factor 2 (SOX2) differentiates HCII from HCI, although supporting cells also express it (Lu et al., 2019; Stone et al., 2021). For HCI, cell morphology was the only identification criterion (Desai et al., 2005a, b) until discovery of molecular components of the calyceal junction that adheres the calyx afferent with the cell, namely, contactin-associated protein (CASPR1) and tenascin-C (Sousa et al., 2009; Lysakowski et al., 2011; Sedó-Cabezón et al., 2015). Only recently Mc Inturff et al. (2018) identified one positive HCI marker, osteopontin, also known as secreted phosphoprotein 1 (SPP1). Using osteopontin for HCI and MAPT and ANXA4 for HCII, these authors characterized the spatial and temporal development of HCI and HCII in the mouse utricle. They reported substantial postnatal maturation of the HCs, in agreement with previous studies (Rüsch et al., 1998; Burns et al., 2012; Warchol et al., 2019).

The postnatal maturation of the rat and mouse vestibular HCs and their fragility to experimental manipulation are significant factors that condition the establishment of *in vitro* models to study their biology and pathology. In one popular preparation, utricles are obtained from adult mice and are maintained as free floating specimens for a few (up to 7, usually 1 to 4) days (Cunningham et al., 2002; Cunningham, 2006; Sung et al., 2022). These explants have been frequently used to study ototoxicity and to evaluate otoprotective treatments in mature HCs, but culturing the sensory epithelium causes a cellular stress that probably masks the earliest responses to the toxicity being analyzed. Another preparation is that of perinatal rat and mouse utricles obtained from embryonic day 19.5 (E19.5) to postnatal day 4 (P4) and cultured in adherent conditions (Werner et al., 2012; 2020; Wang et al., 2015). These explants have been used for short, but also for long (up to 28 days) periods that allow the HCs to mature. However, this seems to be associated with increasing stress and decreasing viability. A common limitation of both these models is that the apical surface of the explant is covered by culture medium, which differs significantly from the endolymphatic fluid to which the surface of the HCs is exposed *in vivo*. One interesting alternative is that of 3D cultures, in which the non-sensory epithelium surrounding the sensory patch grows until it closes a cavity that mimics the endolymphatic compartment existing *in vivo*. The formation of these 3D explants, named cysts, has been reported to depend on the presence of extracellular matrices, either matrigel (Gaboyard et al., 2005; Brugeaud et al., 2007; Bartolami et al., 2011; Tallandier et al., 2021; Tallandier et al., 2023) or collagen-based gels (Gnedeva et al., 2018).

In the present study, we present an updated description of the HC type composition of the vestibular epithelia of the adult rat obtained by counting all the HCs in confocal microscopy images comprising the entire sensory organ. We also present a description of the post-natal maturation of HCI and HCII according to the expression of specific molecular markers. Finally, we present a new model for long term 3D culture of these epithelia that replicates the *in vivo* maturation process.

## 2 Methods

### 2.1 Animals

Adult male and female Long-Evans rats (8–9 weeks old) were purchased from Janvier (le Genest Saint Isle, France). Animals were used directly or mated at the local animal facility to obtain descendants. For this purpose, additional pregnant females were also obtained from the same supplier. Eight rats (6 female, 2 male) were used for the characterization of the HCs in the adult sensory organs. Each estimate was obtained on several organs from at least two different animals as described throughout the text. To characterize the developmental maturation of the HCs *in vivo*, 24 newborns from three litters from different progenitors were used for histology at postnatal days 0 (P0), P1, P4, P7, P10, P14 or P28. Other newborns were used at postnatal day 1 (P1) for tissue culture. The present *in vitro* data were obtained on tissues from 57 pups taken from 12 litters. For tissue collection, rats were euthanatized by decapitation, after prior anesthesia of P14 and older animals. For direct histology, temporal bones were quickly immersed in fixative (4% paraformaldehyde in phosphate buffered saline (PBS), pH 7.2) and the vestibular epithelia were dissected using a binocular microscope under a fume hood. The tissues were fixed for 1 h in the same fixative, then rinsed twice for 20 min in PBS and immersed in a cryopreservative solution (34.5% glycerol, 30% ethylene glycol, 20% PBS, 15.5% distilled water) for storage at −20°C until further processing (Maroto et al., 2021).

### 2.2 Culture of vestibular sensory epithelia

Vestibular epithelia were collected from P1 rats. The dissection was performed in Leibovitz’s L-15 medium (Gibco, cat. # 11415064) in sterile conditions. Otoconial membranes were removed from utricles and saccules and vestibular nerves were trimmed, but the membranes roofing the endolymphatic spaces were not. The epithelia were then cultured free floating for up to 27 days (referred as to 28 DIV, corresponding to P1+ 27 days in culture) in a standard culture medium composed of Dulbecco’s Modified Eagle’s Medium-Nutrient Mixture F12 (DMEM/F12) (Gibco, cat. #11320033) supplemented with 25 mM HEPES (Gibco, cat.# 15630080), 2% N2 supplement (Cell Therapy Systems, cat.# A1370701), 1% GlutaMAX (Gibco, cat.# 35050061), 2 g/L glucose (Sigma, cat.# 49163) and 1.5 g/L penicillin (ThermoFisher, cat.#J63032.14), as described for adult mouse utricle cultures (Cunningham, 2006; Rua et al., 2013). The culture plates were incubated in a 5% CO_2_/95% air environment at 37°C. In one experiment, several modifications to enrich these standard conditions were evaluated for their potential to increase HC survival, as hypothesized from literature data (Koeler et al., 2017; Meas et al., 2018; Lou et al., 2023). These were the addition of 1% Matrigel (Corning, cat.# 356237), the simultaneous addition of 1% -B27 (Gibco, cat. # 17504044) and 50 ng/mL mouse recombinant EGF (Gibco, cat.# PMG8041), and the application of slow orbital rocking (5 rmp, 7°, using a Biosan 3D Sunflower mini-shaker). These modifications were applied single or in combination for the entire period (28 days) or for the first half (days 1–14) of the period only. In another experiment, the culture medium was enriched for the four initial days only (DIV4) with growth and protectant factors. Thus, the standard medium was enriched with 50 ng/mL EGF (Ding et al., 2016; Preprotech, cat.# AF100-15), 0.1 μg/mL FGF2 (Jeong et al., 2021; Sigma, cat.#F0291) and 0.1 μg/mL FGF10 (Zelarayan et al., 2007; Preprotech, cat.# AF-100-26), as well as with 1 mM N-acetyl-L-cistein (Sigma, cat.#A9165), 10 mM nicotinamide (Sigma, cat.#N0636) and 1% insulin-transferrin-selenium-ethanolamine (ITS-X) supplement (Gibco, cat. # 51500056). The enriched medium was changed at DIV1 and DIV3 and filtered at DIV2 and DIV4. From DIV5 onwards, the cysts were maintained in standard medium, changed every other day. For histological assessment, vestibular cultures were fixed and stored as described above.

### 2.3 Immunohistochemistry

Whole-mount immunohistochemical labeling of the vestibular epithelia was performed using 3 or 4 of the primary antibodies listed in table 1, and suitable combinations of secondary antibodies conjugated with Day-Light-405 or Alexa-405, Alexa-488, Alexa-555 and Alexa-647 fluorophores (Invitrogen), according to published protocols (Lysakowski et al., 2011; Maroto et al., 2021a,b). In brief, the specimens were first rinsed with PBS and then permeated and blocked with 4% Triton-X-100% and 20% donkey serum in PBS for 1 h at room temperature. Next, they were incubated with the primary antibody mixture containing 1% donkey serum and 0.1% Triton-X-100 in PBS, for 24 h at 4°C. Secondary antibodies were incubated overnight in the same conditions plus protected from light. The specimens were rinsed with PBS between each incubation step and gently rocked at all steps. Fluoromount (Sigma-Aldrich cat.#F4680) was used for whole-mounting the samples.

**TABLE 1:** Primary antibodies.

Target	Dilution	Host and type	Source	Specificity
Calretinin	1/500	Guinea pig polyclonal	214.104, Synaptic Systems, RRID: AB_10635160	Co-localizes with anti-calretinin antibodies validated in KO mice (CR 7699 from Swant)
Contactin-associated protein 1 (CASPR1)	1/400	Mouse monoclonal (IgG1)	Clone K65/35, UC Davis/Neuromab, RRID: AB_2083496	Western blot on rat brain membranes from control and caspr1-KO mice (Datasheet). See also Sousa et al. (2009), Lysakowski et al. (2011), Sedó-Cabezón et al. (2015)
Myosin 7A	1/100	Mouse monoclonal, IgG1	138-1-s, Developmental Studies Hybridoma Bank, RRID: AB_2282417	Validated by immunoblot and differential tissue expression Hasson et al. (1997). Widely used for specific hair cell labelling
Myosin 7A	1/400	Rabbit polyclonal	25-6790, *Proteus* Biosciences, RRID: AB_10015251	Validated by immunoblot, widely used for specific hair cell labelling. See Pujol et al. (2014)
Myosin 7A	1/200	Rabbit polyclonal	PA1-936, Invitrogen, RRID: AB_2235704	Validated by western blot and relative expression analysis (datasheet). Shown to label cochlear hair cells Xu et al. (2021)
Ospeopontin (Spp1)	1/200	Goat polyclonal	AF808, RD Systems, RRID: AB_2194992	Specifically labels type I hair cells as described McInturff et al. (2018), in perfect concordance with CASPR1 label of encasing calyces Sousa et al. (2009)
Plasma membrane calcium-transporting ATPase 2 (PMCA2)	1/400	Rabbit polyclonal	PA1-915, Invitrogen, RRID: AB_2243199	Validated by immunocytochemistry of control and knockdown cells (Datasheet)
Sex-determining region Y-box 2 (SOX2)	1/200	Mouse monoclonal, IgG1	Clone E−4, sc-365823, Santa Cruz Biotechnology	Validated by western blot (datasheet) and widely used. RRID:AB_10842165

### 2.4 Confocal imaging and cell counts

The vestibular epithelia were imaged in a Zeiss LSM880 spectral confocal microscope. The tile scan function was used to obtain images comprising the entire epithelium with a ×40 objective (NA 1.3). The scan was adjusted to acquire images with a 10% overlap thus facilitating the accurate construction of the merged image. From these images, we obtained the number of HCs showing one specific label or one particular combination of labels in the complete epithelium by manually marking them using the Multipointer function or the Cell Counter plugin of the ImageJ software (National Institute of Mental Health, Bethesda, Maryland, United States). The images of epithelia in the figures are shown in pseudocolors selected to be accessible to people with colorblindness using the David Nichols’ website (https://davidmathlogic.com/colorblind/#%23F93535-%234071F9-%23FFFF07-%234FEF17).

### 2.5 Data analysis and statistics

Data are shown as mean ± SE or as individual points. The same data are also provided in table format in the Supplementary Table File. Group comparisons were made by the Student’s t-test or one-way ANOVA, as appropriate, using the GraphPad Prism 9 software. The Tukey test was used for multiple comparisons after significant ANOVA. The alpha error level was set at 0.05.

## 3 Results

### 3.1 Cellular composition of the vestibular epithelia of the adult rat

**FIGURE 1:**
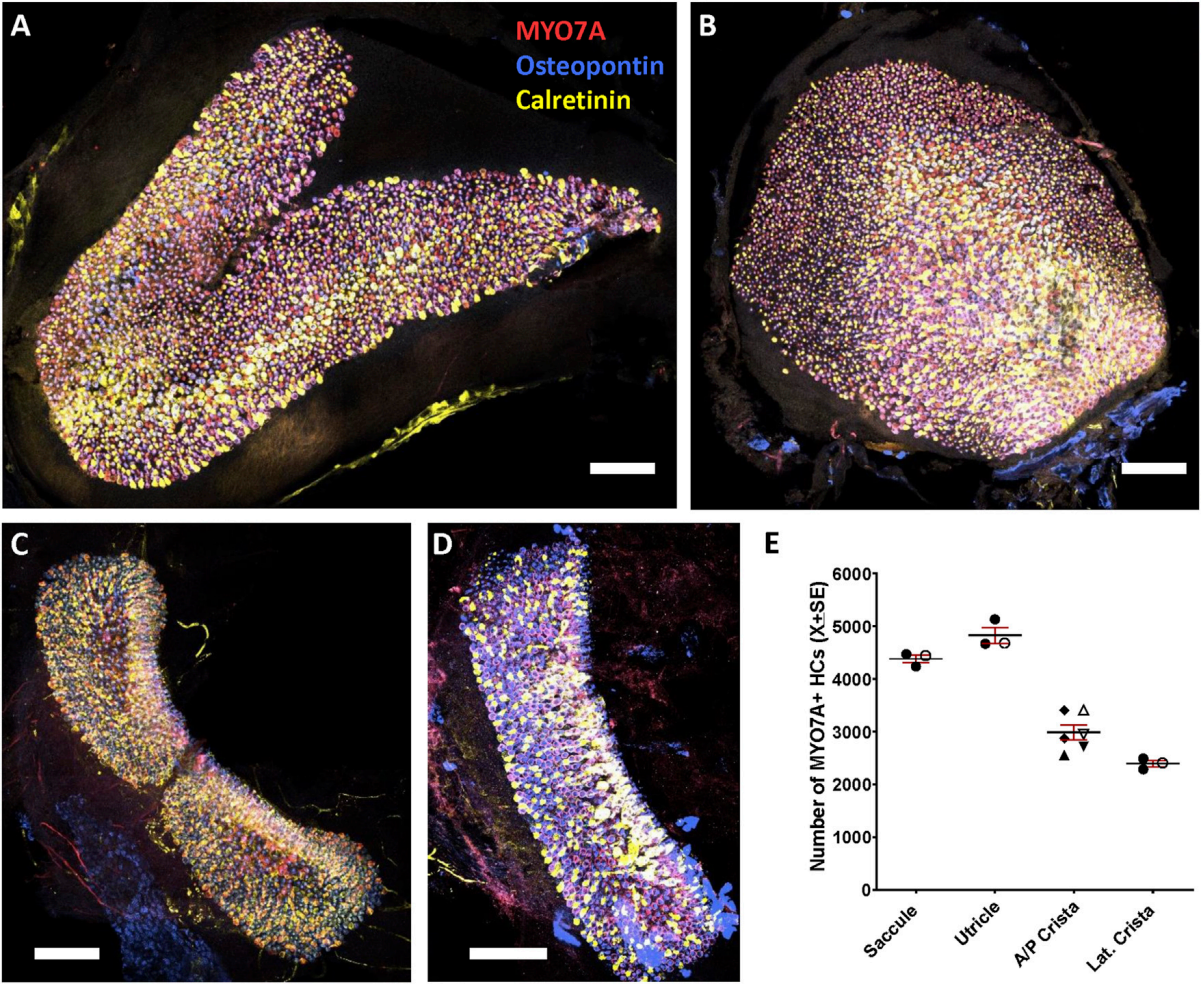
Hair cells (HC) in the vestibular saccule **(A)**, utricle **(B)**, anterior/posterior crista **(C)**, and lateral crista **(D)** of the adult Long-Evans rat. Whole-mount epithelia were immunolabelled with antibodies against MYO7A (138-1-s, shown in red), osteopontin (AF808, shown in blue) and calretinin (214.104, shown in yellow), then imaged by tile scan in a confocal microscope. Shown here are maximum intensity projections from several non-consecutive optical planes, selected for display. The entire stack was used for HC counting. Scale bars = 100 μm. Note that the lateral crista in D is displayed at higher magnification than the epithelia in **(A–C)**. Higher magnification images and separate colour channels are shown in Figure 2. **(E)**. Total number of MYO7A + HCs in the saccule, utricle, anterior/posterior (A/P) crista, and lateral (Lat.) crista. Graph displays mean ± SEM and individual values. Epithelia were derived from three female (solid symbols) and one male (open symbols) animals. The A/P crista include anterior cristae (up-right triangles), posterior cristae (inverted triangles) and unidentified anterior/posterior cristae (diamonds).

Total numbers of HCs in the vestibular epithelia of young adult Long-Evans rats were determined using specimens immunolabelled with antibodies against MYO7A, osteopontin and calretinin. MYO7A is a well described marker of all HCs (Hasson et al., 1997) and by counting MYO7A + cells we obtained the following mean numbers: 4827 in the utricle, 4384 in the saccule, 2987 in the anterior/posterior crista and 2395 in the lateral crista (Figure 1). These mean values were from one male and two female rats and the counts from the three animals were in the same range. Also, in two of the three animals examined, the anterior and posterior crista were identified, and no difference in cell numbers was apparent between these sensory organs (rat 1: anterior 2556, posterior 2721; rat 2: anterior 3409, posterior 2951).

**FIGURE 2:**
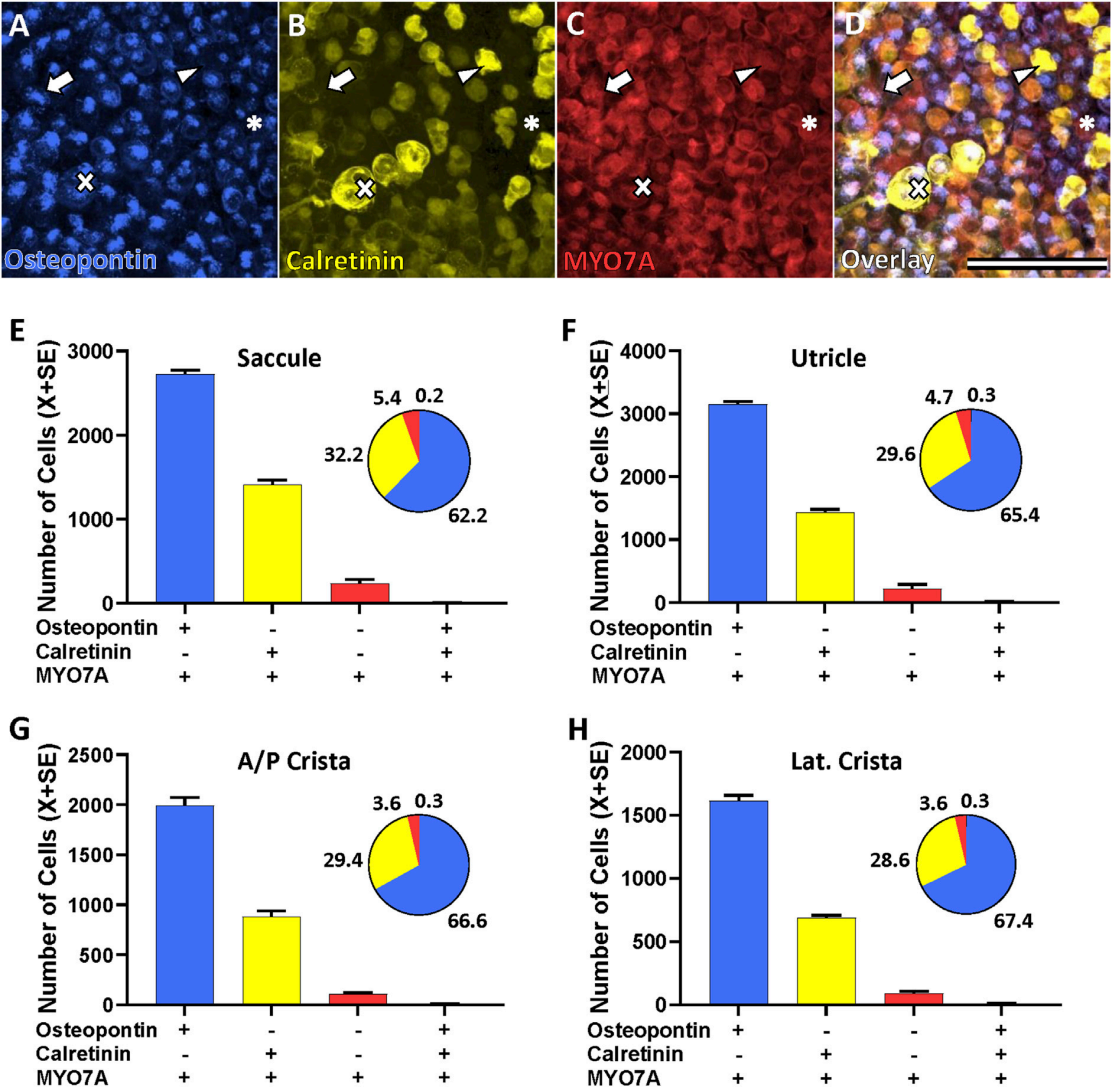
Hair cell (HC) types in the vestibular epithelia of the adult Long-Evans rat as defined by the expression of molecular markers. **Top.** Confocal microscopy images showing the immunolabelling of an utricle with antibodies against osteopontin (AF808, **(A)**, calretinin (214.104, **(B)**, and MYO7A (138-s-1, **(C)**. Panel **(D)** shows the overlap of the three markers. Scale bar = 50 μm. **Arrows:** type I HCs (HCI) expressing osteopontin but not calretinin. **Arrowheads:** type II HC (HCII) expressing calretinin but not osteopontin. **Asterisks:** MYO7A-only HCII, expressing neither osteopontin nor calretinin. **Crosses:** HCI expressing osteopontin encased by a calyx from a calyx-only afferent that expresses calretinin. **Bottom.** Counts of the HC types according to their expression of these markers in the saccule **(E)**, utricle **(F)**, anterior/posterior crista **(G)**, and lateral crista **(H)**. Bar graphs show mean number of HCs ± SE (*n* = 6 anterior/posterior crista; *n* = 3 other epithelia). Pie charts show the percentages of each cell type in the corresponding epithelium.

Figure 2 shows the cell counts using osteopontin and calretinin as molecular markers. Only 0.2%–0.3% of the MYO7A+ HCs were counted to express both osteopontin and calretinin, indicating that these two markers identify two different populations of HCs. Therefore, in the adult rat, differently from neonates, MYO7A+ HC that express osteopontin do not express calretinin, and those that express calretinin do not express osteopontin. Besides these two populations, a third sizeable cell population was that of HCs expressing neither one of these two markers, but only the pan-HC marker MYO7A (MYO7A-only HCs). The proportion of each of these 3 cell kinds was alike in all types of sensory epithelia and the overall percentages from the 52,738 HCs identified were 65.3% osteopontin+ HCs, 30.1% calretinin+ HCs, and 4.3% MYO7A-only (osteopontin-, calretinin-) HCs.

**FIGURE 3:**
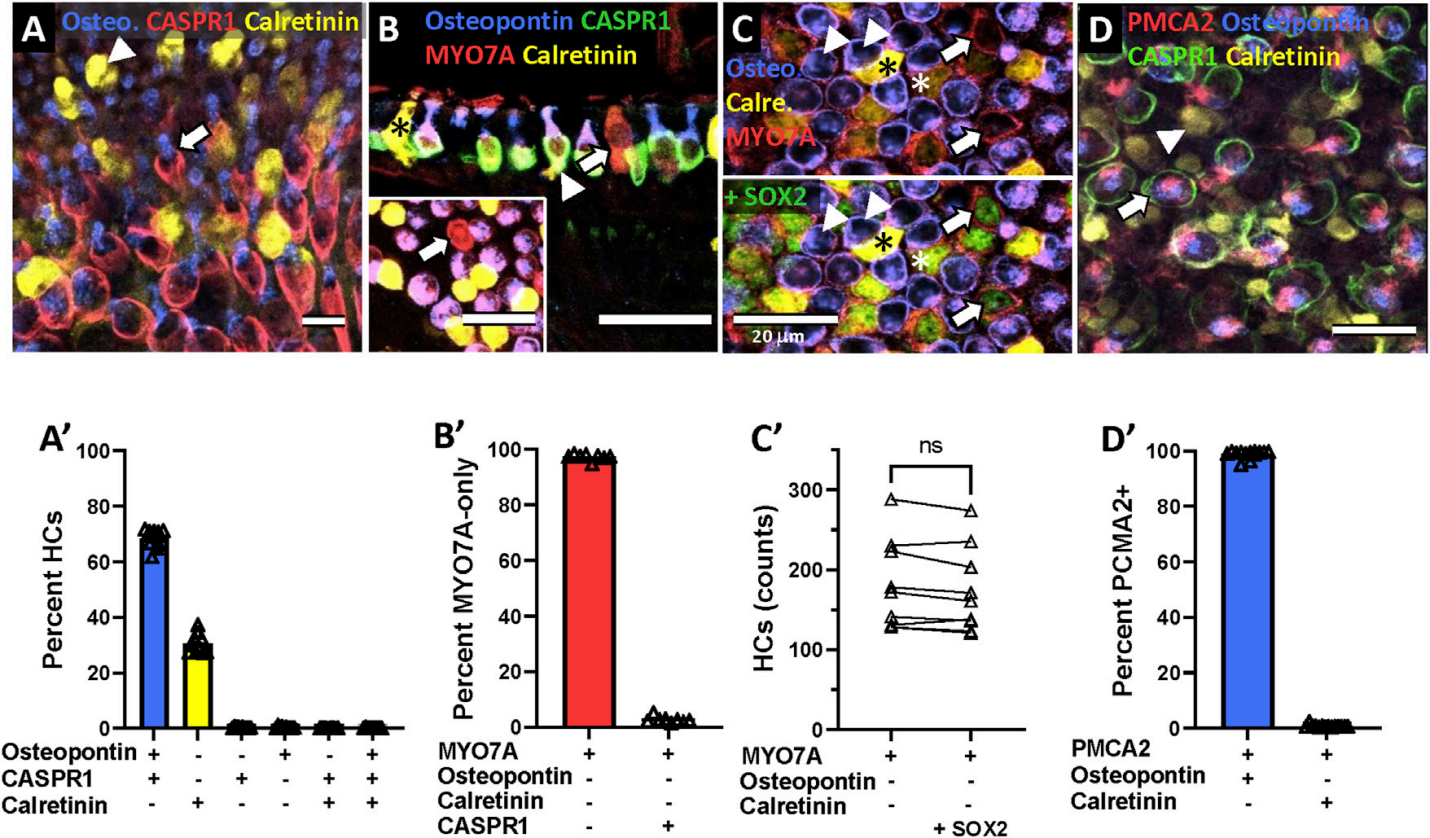
Specificity of the molecular markers for hair cell (HC) types in the vestibular epithelia of the adult Long-Evans rat. **Images in top row:** Confocal microscopy images illustrating the immunolabelling results. **Graphs in bottom row:** Quantitative analyses. **(A)** Arrow: HCs that express osteopontin (blue) are associated with calyces that express CASPR1 (red). Arrowhead: HCs that express calretinin (yellow) are not associated with CASPR1+ calyces. **(B)** Arrow: HCs that express MYO7A (red) but neither osteopontin (blue) nor calretinin (yellow) are not associated with a CASPR1+ calyx (green in the main panel). Asterisk: HCII expressing calretinin. Arrowhead: example of HCI with high expression of osteopontin above the nucleus (blue) and CASPR1+ calyx (green) encasing the basolateral part of the cell. **(C)** In the top half-pannel, arrows point to HCs (MYO7A+, red) that do not express neither osteopontin (blue) nor calretinin (yellow). In the bottom half-pannel, the same image is displayed with the green channel activated to show SOX2 label. Arrowheads: examples of HCI with MYO7A (red) and osteopontin (blue) label. Two HCII expressing intense (black asterisk) or weak (white asterisk) calretinin label are also positive for SOX2. **(D)** Arrow: Stereocilia labelled with the PMCA2 antibody (red) arise from HCs that express osteopontin (blue) and are surrounded by a CASPR1+ calyx (green). Arrowhead: Stereocilia of HCs expressing calretinin (yellow) do not express PMCA2. Scale bars: A, 10 μm; B-main, 30 μm; B-inset, C and D, 20 μm. **(A′-D′)** Quantitative analyses. Data in A′, B′ and D′ are the percentage of HCs showing the different possible combinations of markers in the HC or the associated calyx, as indicated on the abscissa. Each individual point is the percentage obtained in an individual vestibular epithelium (crista, utricle or saccule). Bars are mean values of these points. Data in C′ are absolute counts per epithelium of cells showing a MYO7A+/osteopontin-/calretinin-profile or the same profile plus SOX2 label. Data in A′ are from 10 epithelia from two different animals, those in B′ are from eight epithelia from two animals, those in C′ are from nine epithelia from three animals and those in D′ are from 14 epithelia from four animals. ns: Non-significant at *p* = 0.05, paired *t*-test.

Osteopontin has been described as good marker for HCI in the mouse (McInturff et al., 2018) while calretinin has been reported to identify a large but somewhat variable proportion of HCII in several species (Dechesne et al., 1991; Desai et al., 2005a; Desai et al., 2005b; McInturff et al., 2018). To corroborate their cell type specificity in the adult rat, we determined their association with calyx afferents, identified by the calyx junction adhesion protein CASPR1 (Sousa et al., 2009; Lysakowski et al., 2011; Sedó-Cabezón et al., 2015). As shown in Figures 3A–A', the data (*n* = 26,343 HCs from 10 epithelia derived from 2 different animals, one male and one female) clearly demonstrated the association of osteopontin + cells with CASPR1+ calyces (67.3%), whereas calretinin+ cells are not encased by calyces (31.8%). Other possible combinations of these three markers resulted in very small counts (<0.3% each). Therefore, osteopontin and calretinin selectively label HCI and HCII, respectively, in the adult rat.

Besides the osteopontin+ HCI and the calretinin+ HCII, we aimed to reveal the identity of the MYO7A-only HCs, that is, those expressing neither osteopontin, nor calretinin. Two approaches were used. First, we used the association with CASPR1+ calyces as a criterion to identify *bona fide* HCI. In epithelia labeled with anti-MYO7A, anti-osteopontin, anti-calretinin and anti-CASPR1 antibodies, the cell counts demonstrated that the MYO7A+/osteopontin-/calretinin- HCs do not associate with CASPR1+ calyces (Figure 3B-B′, *n* = 1,202 HCs, from 8 epithelia from 2 different animals), indicating that they are not HCI. In agreement with this observation, their cellular shape matched the known features of HCII (Figure 3B, arrow). The second approach to confirm this identity was to examine whether these cells express the transcription factor SOX2, demonstrated to determine HCII identity (Stone et al., 2021). In epithelia labeled with anti-MYO7A, anti-osteopontin, anti-calretinin and anti-SOX2 antibodies, we counted the number of MYO7A+/osteopontin-/calretinin- HCs per epithelium, and compared it with the number of MYO7A+/osteopontin-/calretinin-/SOX2+ cells. As shown in Figure 3C-C′, the same numbers were obtained when the SOX2 label was added to the MYO7A+/osteopontin-/calretinin- identification (paired *t*-test, t_8df_ = 2.23, ns, *n* = 9, one utricle, one saccule and one crista each from 3 different animals). This confirmed that most or all of the MYO7A+/osteopontin-/calretinin- HCs also express the positive HCII marker SOX2. Therefore, these MYO7A+/osteopontin-/calretinin- HCs are a subpopulation of HCII that do not express calretinin.

Other research in the laboratory suggested that the plasma membrane calcium-transporting ATPase 2 (PMCA2), which is expressed in the stereocilia (Yamoah et al., 1998), could be selective for HCI. We therefore evaluated whether PMCA2 associated with either osteopontin or calretinin expression. The data, shown in Figure 3D-D′, demonstrated that HCI, not HCII, express PMCA2 in their stereocilia bundles (*n* = 32,064 PMCA2+ HCs from 3 saccules, 3 utricles, 2 lateral crista and 6 anterior/posterior crista, derived from 2 male and 2 female rats). Thus, PMCA2 is also a specific marker of HCI.

### 3.2 Maturation of HC types during the postnatal period

**FIGURE 4:**
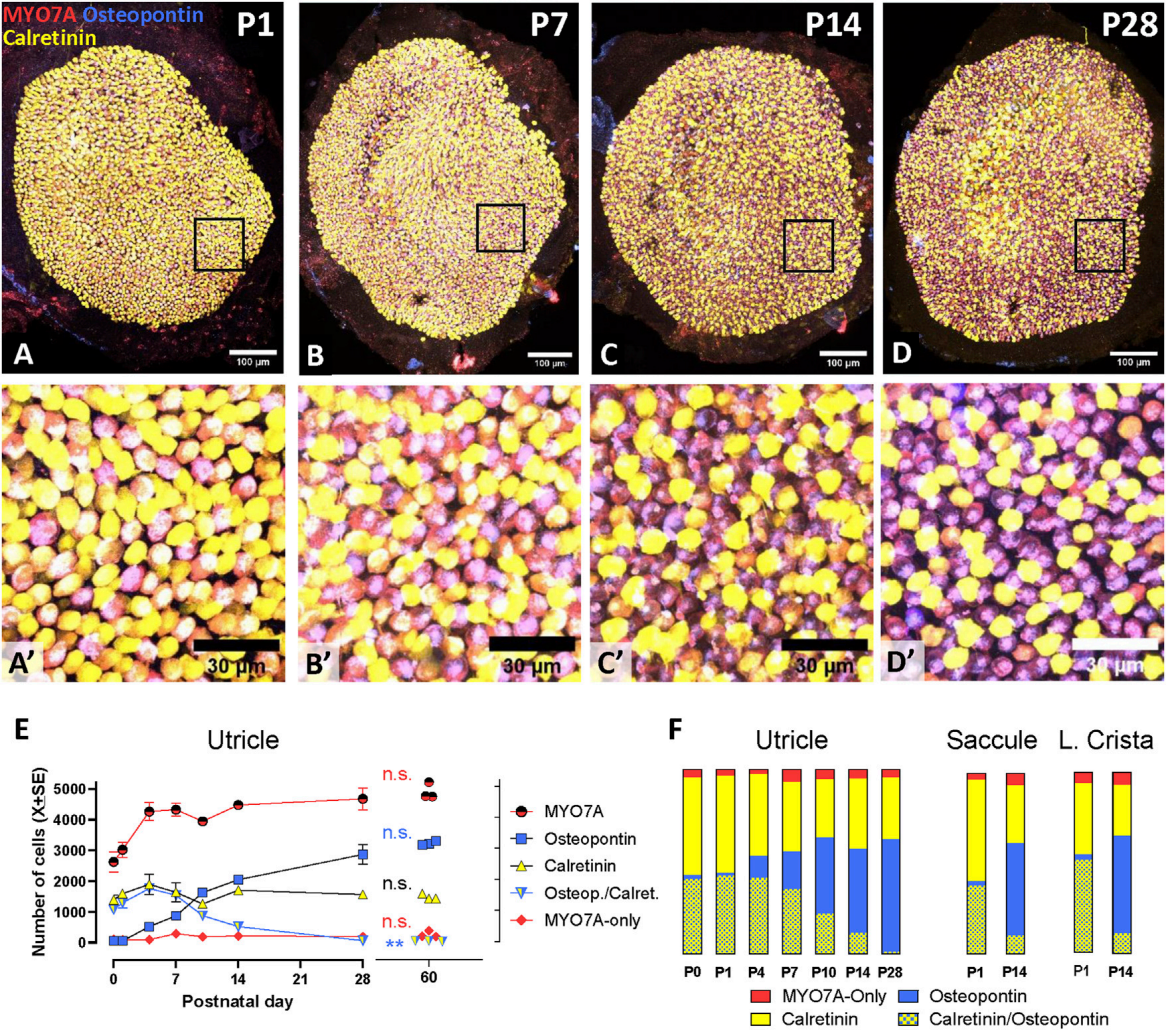
Maturation of the vestibular sensory epithelia of Long-Evans rats from birth (P0) to postnatal day 28 (P28). **Top (A–D; A′-D′)**. Confocal microscopy images showing utricles immunolabelled with antibodies against MYO7A+ (red), osteopontin (blue), and calretinin (yellow) at postnatal days 1, 7, 14, and 28. The first row shows the entire utricles. Higher magnification images of the boxed areas in the first row are shown in the second row. Scale bars: A-D, 100 μm; A′-D′, 30 μm. **Bottom.** Quantitative analyses. **(E)**. The graph displays the mean (±SE, when larger than point size) numbers of cells of each immunolabelling type at each time point in the utricle (*n* = 3/time point). Replicates are from three different rats from at least 2 litters from different progenitors, except at day 10, at which all rats were siblings. The data from adult rats, already shown in Figure 2.F, are included for comparison. **n.s.**: No statistically significant difference between P28 and P60 data. **: Statistically significant difference (*p* < 0.01) between P28 and P60 in the number of immature HCs expressing both osteopontin and calretinin. **(F)**: Bars show the percent distribution of HCs at each postnatal day in the utricle (data derived from data in panel I), and at P1 and P14 in the saccule and the lateral crista. From top to bottom, the sectors show the percentage of HCs expressing neither osteopontin nor calretinin (MYO7A-only, about 10%–15% of the HCII in adult rats), calretinin (85%–90% of the HCII in adult rats), osteopontin (HCI), and both osteopontin and calretinin (immature HCs).

Significant part of the maturation of the rodent vestibular epithelium occurs during the postnatal period (Nordemar, 1983; Rusch et al., 1998; McInturff et al., 2018). To describe the maturation process with the selective molecular markers used above for the adult tissues, we obtained cell counts in epithelia from postnatal day 0 (P0) to P28 (Figure 4). A detailed time course analysis was performed in the utricle (Figures 4A–E). The total number of MYO7A+ HCs increased during the first postnatal week and remained stable thereafter. At P0, very few HCs expressed only osteopontin, while about 40% of the cells showed an immature phenotype and expressed both osteopontin and calretinin. The number of these osteopontin+/calretinin+ cells declined with time and that of HCs expressing only osteopontin increased largely. Interestingly, the small population of MYO7A cells expressing neither calretinin nor osteopontin previously identified in adult rats was similarly present at all postnatal time points. At day 28, the utricle displayed a nearly mature composition, and no significant differences were found between P28 and P60 utricles in most parameters: total number of HCs (t_4df_ = 0.575; *p* = 0.596), number of osteopontin + HCI (t_4df_ = 1.266; *p* = 0.274), number of calretinin + HCII (t_4df_ = 2.062; *p* = 0.108), and number of MYO7A-only HCII (t_4df_ = 0.465; *p* = 0.566). However, a small (1.3%) but significant (t_4df_ = 5.139; *p* = 0.007 compared to P60) number of immature HCs, expressing both osteopontin and calretinin, was still present in the P28 utricles.

HC percentages were obtained from total cell counts assessed in P1 and P14 saccules and lateral cristae (Figure 4F). Like the utricles, these epithelia showed at P1 a large percentage of immature HCs expressing both osteopontin and calretinin. At this stage, comparison of the percentages of cell types among the three epithelia indicated that the crista had a significantly larger proportion of immature cells than the saccule (52% *versus* 38%, *p* < 0.05) and a smaller proportion of calretinin cells than both the saccule and the utricle (39% *versus* 56% and 53%, respectively, *p* < 0.05). However, these differences were reduced with time, and the only difference found at P14 was in the percentage of osteopontin cells, smaller (*p* < 0.05) in the utricle (46%) than in the crista (54%). Similarly to the utricle, HCs expressing osteopontin and not calretinin (i.e., HCI) were already the most abundant cell type at this time point in both the saccule and the lateral crista.

### 3.3 *In vitro* 3D culture and maturation of vestibular sensory epithelia

**FIGURE 5:**
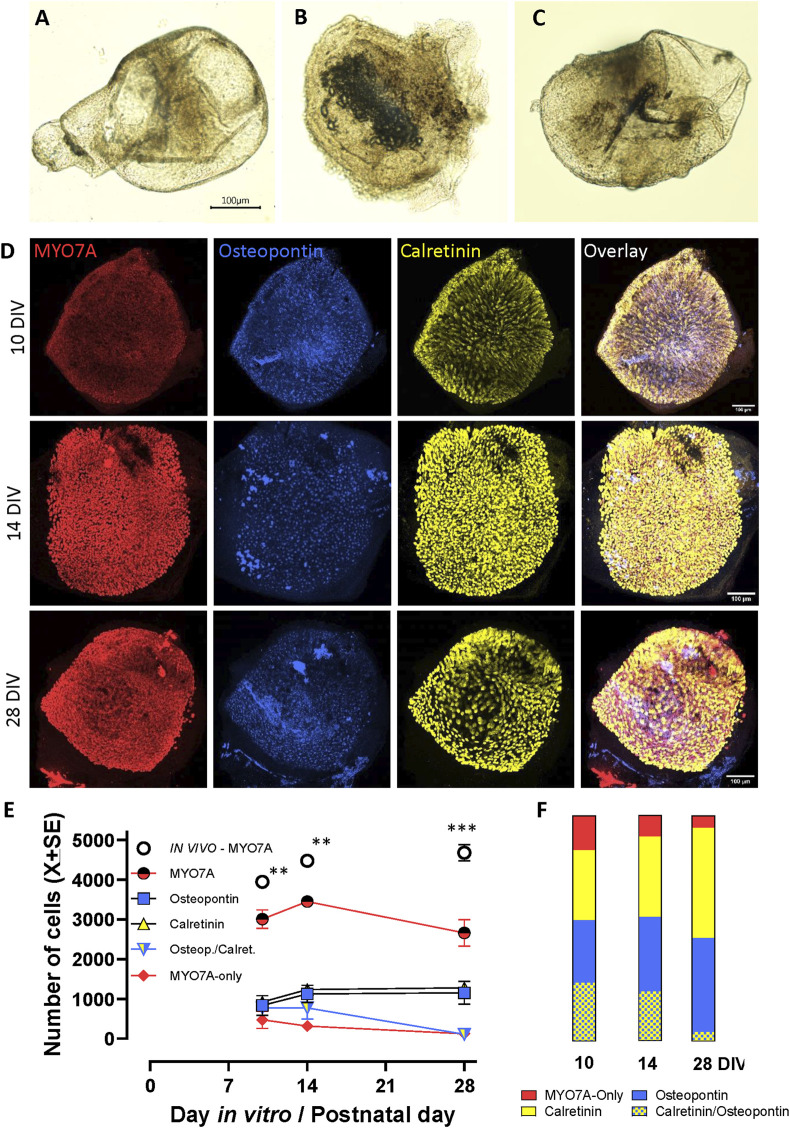
3D culture and maturation of vestibular sensory epithelia of the rat. Cristae, utricles and saccules were obtained at postnatal day 1 (P1) and cultured free floating in standard culture medium for up to 27 additional days (28DIV). (**A–C)**. Aspect of the crista, utricle and saccule, respectively, at 14 DIV. Note that each sensory epithelium is included in a cyst with a non-sensory epithelium enclosing a fluid-filled cavity. **(D)**. Confocal microscopy images of utricles collected at 10, 14 or 28 DIV and immunolabelled for MYO7A (red, first column), osteopontin (blue, second column), and calretinin (yellow, third column). Overlay images are shown in the fourth column. Scale bars: 100 μm. **(E, F)**. Quantitative analysis. **(E)**. Number (mean ± SE, when larger than point size) of each hair cell (HC) type in these utricles (n = 3/time point). The total number of HCs (MYO7A+) in utricles grown *in vivo*, as reported in Figure 4, are included for comparison purposes. **: *p* < 0.01, ***, *p* < 0.001, *in vivo* vs. *in vitro* total HCs. **(F)**. Percent distribution of HCs at 10, 14 and 28 DIV. From top to bottom, the sectors show the percentage of HCs expressing neither osteopontin nor calretinin (MYO7A-only, about 10%–15% of the HCII in adult rats), calretinin (85%–90% of the HCII in adult rats), osteopontin (HCI), and both osteopontin and calretinin (immature HCs).

In preliminary experiments, we observed that most vestibular epithelia obtained at P1, including utricles, cristae, and saccules, successfully closed a fluid-filled cavity within 2–3 days of placement in free-floating conditions into standard culture medium. Cyst formation efficiency declined over the first postnatal week and did not require the presence of an extracellular gel (data not shown). Therefore, all subsequent studies were done with free-floating epithelia obtained at P1. For an initial evaluation of the viability of these cysts, we cultured epithelia in these standard conditions until day 28 (28 DIV, meaning P1+ 27 days *in vitro*). As shown in Figures 5A–C, cysts cultured in free-floating conditions in standard culture medium had a macroscopic appearance resembling the morphology of the corresponding epithelium *in vivo*. HC maturation *in vitro* was assessed by comparing utricles cultured for 10, 14, and 28 DIV (Figures 5D–F). The total number of HCs at these days was lower than those obtained at the same days *in vivo* (76%, 77%, and 57%, respectively; *p* < 0.05 in all three comparisons, Figure 5D). However, no significant differences in the number of total HCs occurred when comparing across days 10, 14, and 28 in culture. When HC types were determined (Figures 5E,F), we observed that the numbers of immature HCs, expressing both calretinin and osteopontin declined with time (from 26.0% to 4.1%), while the number of cells expressing only osteopontin or only calretinin increased. At 10DIV, the proportion of HCs expressing only MYO7A (15.0%) was larger than *in vivo*, but this number declined to 5.1% at 28DIV.

### 3.4 Modifications in cyst culture conditions affecting HC differentiation or survival

**FIGURE 6:**
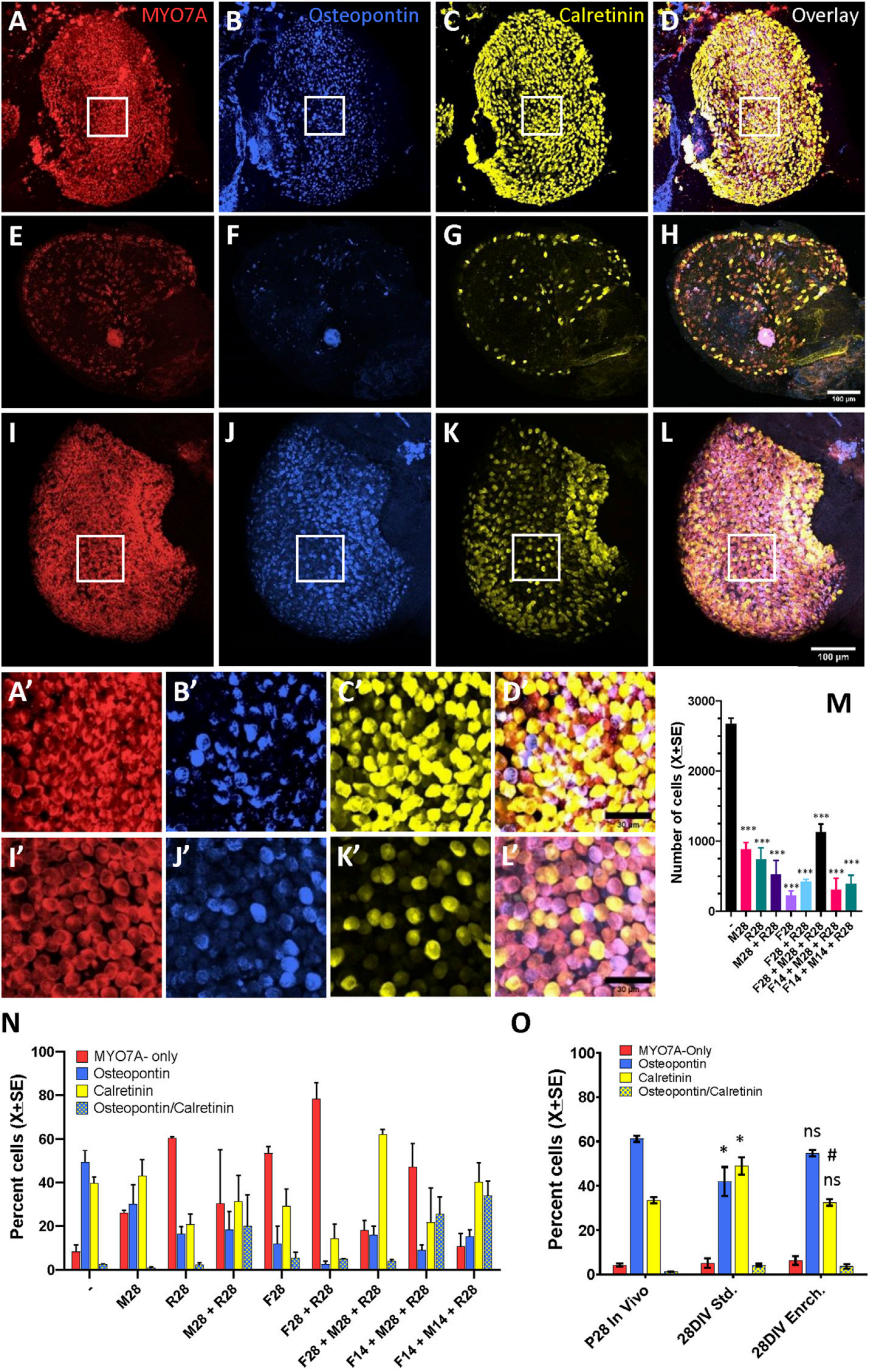
Effect of culture conditions on HC survival and differentiation in 3D cultures of rat vestibular sensory epithelia. Utricles were obtained at postnatal day 1 (P1) and cultured free floating for up to 27 additional days (28DIV). **(A–L)**. Utricles cultured until 28DIV, then immunolabelled for MYO7A (**A, E, I**, red), osteopontin (**B, F, J**, blue), and calretinin (**C, G, K**, yellow). Overlay images are shown in **D, H, L**. **(A–D)**. Utricle cultured in standard medium until 28DIV; note the high density of HCs. **(E–H)**. Utricle cultured under slow orbital rocking in a modified medium containing 1% matrigel, 1% B27 and 50 ng/mL EGF until 28DIV. Note the reduced density of HCs. **(I–L)**. Utricle cultured with an enriched medium (containing EGF, FGF2, FGF10, N-acetyl-L-cistein, nicotinamide and insulin-transferrin-selenin-etanolamine supplement) for 4 days and then in standard medium until 28DIV. Note the high density of HCs and the reduced density of calretinin + HCs. **A′-D′** and **I′-L’.** Higher magnification of the boxed areas of utricles in **(A–D)** and **(I–L)**. Scale bars: 100 mm in A-L, 30 mm in A′-L’. **(M, N)**. Comparison of total HC numbers (**M**) and percentage of HC types according to expression of the molecular markers (**L**) between standard culture conditions (−) and modified culture conditions (M, F, R and their combinations). The modifications were applied from days 1–28 or from days 1–14 only, and the specimens were in both cases collected at 28DIV. The modifications were applied alone or in combination and consisted of 1% matrigel (M), 1% B27 plus 50 ng/mL EGF **(F)**, and slow orbital rocking **(R)**. Data are mean + SEM (*n* = 3/condition). ***: *p* < 0.001, different from standard culture conditions (Tukey’s test after significant ANOVA). **(O)**. Comparison of the percentages of HC types between utricles grown *in vivo* (P28), cultured in standard culture conditions (28DIV Std) and cultured for 4 days in the enriched medium and then in the standard medium until day 28 as described above. *, ns: *p* < 0.05 or non-significant, respectively, in comparison to *in vivo* data. #: *p* < 0.01, different from standard culture conditions. Tukey’s test after significant ANOVA.

We also evaluated the potential improvement of the cyst viability by the addition of several modifications of the standard protocol, alone or in combination for 14 or 28 days. When the effect of 1% Matrigel, orbital rocking (7°, 5 rpm), and trophic enrichment (EGF + B27) were evaluated either alone or combined, the macroscopic appearance worsened in all cases with respect to the appearance shown by cysts maintained in standard conditions. For a quantitative assessment, we immunolabelled utricular cysts to count HCs and their cell type specification. The confocal microscopy images in Figure 6 illustrate the high density of HCs in the 28DIV utricles cultured in standard conditions (Figures 6A–D), and the reduced viability of utricles cultured in enriched conditions (Figures 6E–H). Quantitatively (Figure 6M), utricles maintained in standard medium had 2,688 ± 84 (X±SE) HCs at 28DIV, representing 57% of the total number of HCs obtained at P28 *in vivo* (Figure 4). The number of HCs was significantly reduced by all conditions we evaluated for 14 or 28 days as candidate modifications to improve differentiation and survival (*p* < 0.001 for all conditions, Tukey’s test after significant ANOVA, F (8,18) = 35.54, *p* < 0.001). Comparing the types of cells in the utricles cultured until 28 DIV (Figure 6N), we observed the following proportions for the standard culture conditions: 49.4% osteopontin+, 39.6% calretinin+, 8.4% MYO7A-only, and 2.6% expressing both osteopontin and calretinin. These proportions resembled much more those determined on the P28 utricles *in vivo* (61.2%, 33.4%, 4.4%, and 1.3%, respectively, Figure 4F) than after any of the enriched conditions, in which we observed higher proportions of calretinin+ HCs (up to 62.0% in utricles given the F28/M28/R28 condition) or MYO7A-only HCs, up to 78.3% in F28/R28.

In a second experiment, the culture medium was enriched with several growth and protectant factors for the initial 4 days in culture only, as these are key days for epithelium growth and maturation *in vivo* (Burns et al., 2012). The data obtained showed an improvement in epithelium differentiation (Figure 6O). Thus, the percentages of HCI and calretinin+-HCII (55% and 32%, respectively) in the enriched medium did not differ significantly from the *in vivo* values (61% and 33%), while a significant difference was recorded for the utricle cultures in standard medium for the whole 28 days (42% and 49%, *p* < 0.05 Tukey’s test after significant ANOVA). The ANOVA values were F (2,6) = 6.22, *p* = 0.034 for percent HCI and F (2,6) = 13.39 for percent calretinin+-HCII. Nevertheless, the total number of HCs in the utricles cultured with enriched medium for 4 days (2601 ± 56.8) was similar to the one in utricles cultures with standard medium (2586 ± 299), and both values were significantly lower than the number of HC in utricles from P28 rats (4679 ± 207.9) (Tukey test after significant ANOVA, F (2, 6) = 32.02, *p* = 0.0006).

## 4 Discussion

**FIGURE 7:**
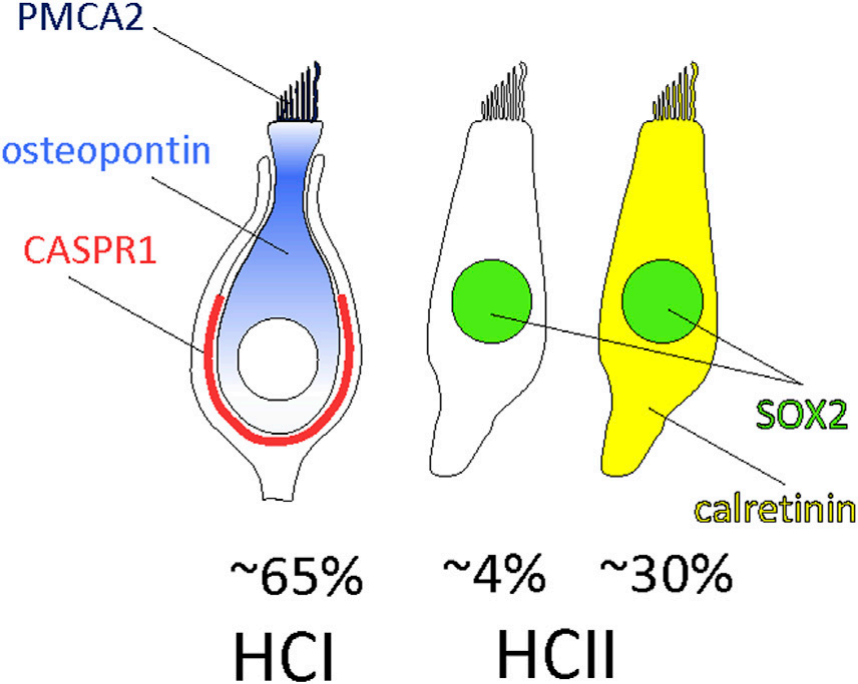
Composition of the vestibular sensory epithelia of the adult rat. Around 65% of the HCs are HCI expressing PCMA2 in the stereocilia and osteopontin in the cytoplasm, more concentrated in the neck to supranuclear zone. They are in contact with calyx afferents expressing CASPR1. The remaining HCs are HCII that express SOX2 in the nucleus. These include 85%–90% HCII expressing calretinin and 10%–15% HCII that do not express calretinin.

The number and types of HCs in the vestibular sensory epithelia have been assessed on several occasions, but some uncertainties remained. The data presented here for the adult rat, summarized in Figure 7, demonstrate that HCI, identified by the CASPR1+ calyx around them, comprise ∼65% of the MYO7A+ HCs and consistently express osteopontin in the cell body and PMCA2 in the stereocilia. The rest of HCs are HCII that express MYO7A and SOX2; these include of a larger population (−30% of all HCs) of HCII that express calretinin and a smaller population (−4%) that do not express calretinin. Using whole 3D images of optimally immunolabelled epithelia we found only very small numbers of HCs not included in this classification, such as HCs labelled with both osteopontin and calretinin or HCs labelled with osteopontin but not surrounded with a CASPR1+ calyx. While previous data from the mouse utricle indicated that osteopontin and calretinin were expressed in most HCI and HCII, respectively (McInturff et al., 2018), the present data indicate that 1) osteopontin and the new marker PMCA2 are highly selective HCI markers in the adult rat, 2) calretinin is a selective marker for 85%–90% of the HCII, 3) MYO7A+ cells that do not express neither osteopontin nor calretinin in the adult are HCII, and 4) identical selectivity and similar proportions are found in crista, utricle and saccule. Whether the MYO7A-only cells represent a true subset of HCII with a particular molecular profile or standard HCII that show no expression of calretinin at the moment of tissue collection remains to be determined. If they were a true subset of HCII, determining their physiological role would be an interesting research objective. Although the possibility that these are another kind of immature HCs cannot be excluded, their presence in the adult weakens this hypothesis.

It is well established that significant maturation of the HCI and HCII occurs during the first postnatal weeks in the mouse utricle (Rüsch et al., 1998; Burns et al., 2012; McInturff et al., 2018; Warchol et al., 2019). The present rat data identifies a large proportion of immature HCs, expressing both calretinin and osteopontin, in the crista, utricle and saccule at P1, and a smaller but still significant percentage of these cells at P14. In the utricles examined a P28, the numbers of total, osteopontin+, calretinin+ and MYO7A-only HCs were similar to those of the adult rat, but a sizeable (1.3%) population of immature cells was still present. Therefore, according to these markers, the maturation of the vestibular HCs is not complete at P14 and extends to the end of the first postnatal month in the rat.

Placement of adult sensory epithelia *in vitro* cause a HC stress that manifests as a significant loss of HCs after several days. Although this damaging effect is often not reported, the data available indicate that it may be quite extensive. For instance, Lin et al. (2011) reported a 66% loss of HCs in adult mouse utricles after 4 days in culture. Conversely, if neonatal epithelia are used for culture and used shortly after plating, a large proportion of HCs are immature as defined by the expression patterns of osteopontin and calretinin. These limitations complicate the interpretation of results from studies of ototoxicity, otoprotection and regeneration for their posterior application in adults. In the present model, using standard culture medium, HCs successfully mature *in vitro*, and this maturation parallels that *in vivo* except for some delay (larger proportion of immature HCs *in vitro* than *in vivo* at P10 [26.0% vs. 22.1%], P14 [22.1% vs. 11.6%] and P28 [4.1% vs. 1.3%]). Also, the stability in total number of HCs between 10DIV and 28DIV suggests that the smaller number of HCs found in 28DIV utricles relative to P28 utricles is due to reduced HC formation after culture respect to the growth that occurs during the first postnatal week *in vivo*, not in a significant occurrence of HC degeneration after *in vitro* maturation.

Using free-floating conditions, a regular spontaneous closure of the cyst is obtained from P1 rats. This demonstrates that early age of the epithelia and not the presence of a gel matrix (Gaboyard et al., 2005; Gnedeva et al., 2018) is the key factor for cyst formation. Although gels are assumed to facilitate the formation of the cysts, they may finally become a stressing factor (for instance, by interfering with oxygen diffusion) for longer culture times (Gaboyard et al., 2005; Brugeaud et al., 2007; Tallandier et al., 2021). In the present free-floating conditions, cysts formed from the three types of vestibular epithelia, cristas, utricles and saccules. This constitutes an improvement with respect to the more frequent use of utricles alone, although at least one other example of crista culture has been published (Tallandier et al., 2023). Notably, the cysts reproduced the *in vivo* morphology (ampullar *versus* macular shape) indicating that this morphology is autonomously determined by the developing vestibule and not shaped by surrounding tissues.

After establishing the basic conditions for epithelial long-term maintenance and maturation *in vitro*, we evaluated several potential modifications of the medium aimed at improving HC survival or differentiation. The modifications expected to provide better long-term conditions (including Matrigel, B27 + EGF and rocking) had a large detrimental effect instead. In contrast, the addition of growth and protective factors (EGF + FGF2 + FGF10 + N-acetyl-cystein + nicotinamide + insulin-transferrin-selenium-ethanolamine) during the initial 4 days in culture did not harm the epithelia (they resulted in numbers of total HCs similar to those in the standard medium) but improved the percentages of HCI and calretinin+-HCII to values near those found *in vivo*.

In summary, the present study fully determined the populations of HCI and HCII in the adult rat using positive molecular markers and characterized the developmental maturation of these markers *in vivo*. It also established a culture protocol for vestibular sensory epithelia that provide good conditions for cyst formation and subsequent HC maturation into differentiated HCI and HCII. The final conditions include an enriched medium for 4 days followed by a standard maintenance medium, resulting in a high yield in HC numbers and a percentage of HCI and calretinin+-HCII near that shown by vestibular epithelia grown *in vivo*. This protocol, open to further improvement, may provide a good model to study HC maturation and survival *in vitro*.

## Data Availability

The raw data supporting the conclusion of this article will be made available by the authors, without undue reservation.
